# Expression-based and co-localization detection of arabinogalactan protein 6 and arabinogalactan protein 11 interactors in Arabidopsis pollen and pollen tubes

**DOI:** 10.1186/1471-2229-13-7

**Published:** 2013-01-08

**Authors:** Mário Costa, Margarida Sofia Nobre, Jörg D Becker, Simona Masiero, Maria Isabel Amorim, Luís Gustavo Pereira, Sílvia Coimbra

**Affiliations:** 1 Departamento de Biologia, Faculdade de Ciências, Universidade do Porto, Edifício FC4 Rua do Campo Alegre, 4169-007, Porto, Portugal; 2 BioFIG, Center for Biodiversity, Functional and Integrative Genomics, Porto, Portugal; 3 Instituto Gulbenkian de Ciência, Oeiras, 2780-901, Portugal; 4 Dipartimento di Biologia, Università degli Studi di Milano, Milan, 20133, Italy

**Keywords:** Arabidopsis, Arabinogalactan proteins, Pollen tube, Microarray, Yeast two-hybrid

## Abstract

**Background:**

Arabinogalactan proteins (AGPs) are cell wall proteoglycans that have been shown to be important for pollen development. An Arabidopsis double null mutant for two pollen-specific AGPs (*agp6 agp11*) showed reduced pollen tube growth and compromised response to germination cues *in vivo*. A microarray experiment was performed on *agp6 agp11* pollen tubes to search for genetic interactions in the context of pollen tube growth. A yeast two-hybrid experiment for AGP6 and AGP11 was also designed.

**Results:**

The lack of two specific AGPs induced a meaningful shift in the gene expression profile. In fact, a high number of genes showed altered expression levels, strengthening the case that AGP6 and AGP11 are involved in complex phenomena. The expression levels of calcium- and signaling-related genes were found to be altered, supporting the known roles of the respective proteins in pollen tube growth. Although the precise nature of the proposed interactions needs further investigation, the putative involvement of AGPs in signaling cascades through calmodulin and protein degradation via ubiquitin was indicated. The expression of stress-, as well as signaling- related, genes was also changed; a correlation that may result from the recognized similarities between signaling pathways in both defense and pollen tube growth.

The results of yeast two-hybrid experiments lent further support to these signaling pathways and revealed putative AGP6 and AGP11 interactors implicated in recycling of cell membrane components via endocytosis, through clathrin-mediated endosomes and multivesicular bodies.

**Conclusions:**

The data presented suggest the involvement of AGP6 and AGP11 in multiple signaling pathways, in particular those involved in developmental processes such as endocytosis-mediated plasma membrane remodeling during Arabidopsis pollen development. This highlights the importance of endosomal trafficking pathways which are rapidly emerging as fundamental regulators of the wall physiology.

## Background

Pollen-pistil interaction is initiated when the male gametophyte is transferred from the anther to the stigma. The pollen grain then starts to hydrate and germinate, forming a pollen tube that grows through the carpel’s internal tissues to deliver its two sperm cells into the embryo sac. Pollen tubes elongate through the extracellular matrix of the pistil tissues, extending by an actin-myosin-based tip-growth mechanism that transports vesicles loaded with new cell wall material to the extending apex and finally, when arriving at the embryo sac, enter into the synergid cell. Fundamentally during this type of localised, tip-focused growth, the cytoplasm is polarized directing secretory events to the tip. Internal gradients and transmembrane ion fluxes, notably of calcium ions, are another key feature of pollen tube growth [1].

Arabidopsis pollen transcriptome analysis showed the expression of a unique subset of genes relative to the sporophytic tissues [2-6]. Furthermore, changes in gene expression patterns occur during the development of the male gametophyte from a microspore to a mature tricellular pollen grain [7]. More recently, genome-wide expression profiling of pollen tubes grown *in vitro* identified another set of genes that are expressed in the pollen tube but not in pollen, which suggests *de novo* mRNA synthesis in the growing pollen tube [5]. The gene expression profiles of *in vitro*- and semi *in vivo*-grown Arabidopsis pollen tubes have also been characterized and were found to differ; this lead to the discovery of a specific sub-set of genes that are activated by potentiation of the pollen tube by the pistil [6].

Among the genes, or gene families that may be involved in the transition from a sporophytic to a gametophytic-developmental program are those which code for arabinogalactan proteins (AGP). AGPs constitute a large family of cell wall proteoglycans that are found on the plasma membrane, in the cell wall, in the apoplastic space, and in secretions. They have also been found in detergent-resistant membranes isolated from Arabidopsis suggesting their presence in lipid rafts [8]. Key distinguishing features of AGPs are: (1) the carbohydrate, usually branched type II arabino-3,6-galactan, *O*-linked to Hyp residues of the protein backbone, which constitutes 90–98% (w/w) of the molecular mass, (2) the protein backbone typically rich in the dipeptide motifs Ala-Hyp, Ser-Hyp, Thr-Hyp, Val-Pro, Gly-Pro and Thr-Pro, (3) a glycosylphosphatidylinositol (GPI) membrane anchor, predicted to be present on most, but not all, AGPs and (4) the ability to bind to a class of synthetic chemical dyes, known as Yariv reagents [9], which are not only useful for detection, quantification and isolation of AGPs, but also for functional studies [10].

The identification of Arabidopsis pollen-specific AGP genes has been used to scrutinize phenotypic changes in the respective null mutants. We previously identified two male gametophyte AGP genes (*AGP6* and *AGP11*) which showed functional redundancy [11]. An *agp6 agp11* double null mutant was subsequently obtained. The double null mutant exhibited segregation distortion, as assessed by the number of aborted pollen grains, suggesting that the gametophyte generation was affected. The strong reduction in pollen germination and pollen tube growth rate, together with premature ectopic germination of pollen (whilst it was still in the anther) [12], prompted us to further analyze this mutant, in order to gain insight into the mode of action of AGPs. We decided to examine the transcriptome of the *agp6 agp11* double null mutant pollen tubes, using the Affymetrix ATH1 Genome Array.

Here we report the identification of 1022 genes whose expression in the double null mutant pollen tubes was shown to be either reduced or elevated, when compared to wild type pollen tubes. These genes can be used as starting points to dissect the gene regulatory networks in which AGPs are involved during pollen tube growth. In parallel, we performed yeast two-hybrid experiments to identify interactors of AGP6 and AGP11, and to provide evidence for the biological functions of these AGPs.

## Results

An Arabidopsis line simultaneously null for two pollen-specific AGP genes, *AGP6* and *AGP11*, has been characterized in our laboratory and found to display notable phenotypic alterations, namely partially aborted pollen grains, reduced germination potential and precocious germination inside the anthers [11,12]. Furthermore, under the conditions used in this study pollen tubes of the double mutant were statistically shorter (83 μm) than those of the wild type (100 μm).

To further characterize the *agp6 agp11* mutant line a differential microarray screen was carried out to identify genes with modified expression in the 8 h-grown pollen tubes of *agp6 agp11* compared to wild type pollen tubes.

### General data analysis

The number of expressed genes in the wild type pollen tubes (as indicated by a minimum of 2 out of 3 Present calls in the triplicate GeneChip experiments) was found to be 6886, which is approximately 28% of the total number of genes represented in the GeneChip Arabidopsis ATH1 Genome Array. This value is in close agreement with those published for microarray studies of Arabidopsis pollen tubes using the same type of microarray chips and experimental set up, i.e., *in vitro*-grown pollen tubes harvested 4–6 h post-germination [5,6]. Microarray data was published in GEO (http://www.ncbi.nlm.nih.gov/geo/info/linking.html) with the record number GSE40861.

### Functional classification of wild type pollen tube transcriptomes

**Figure 1:**
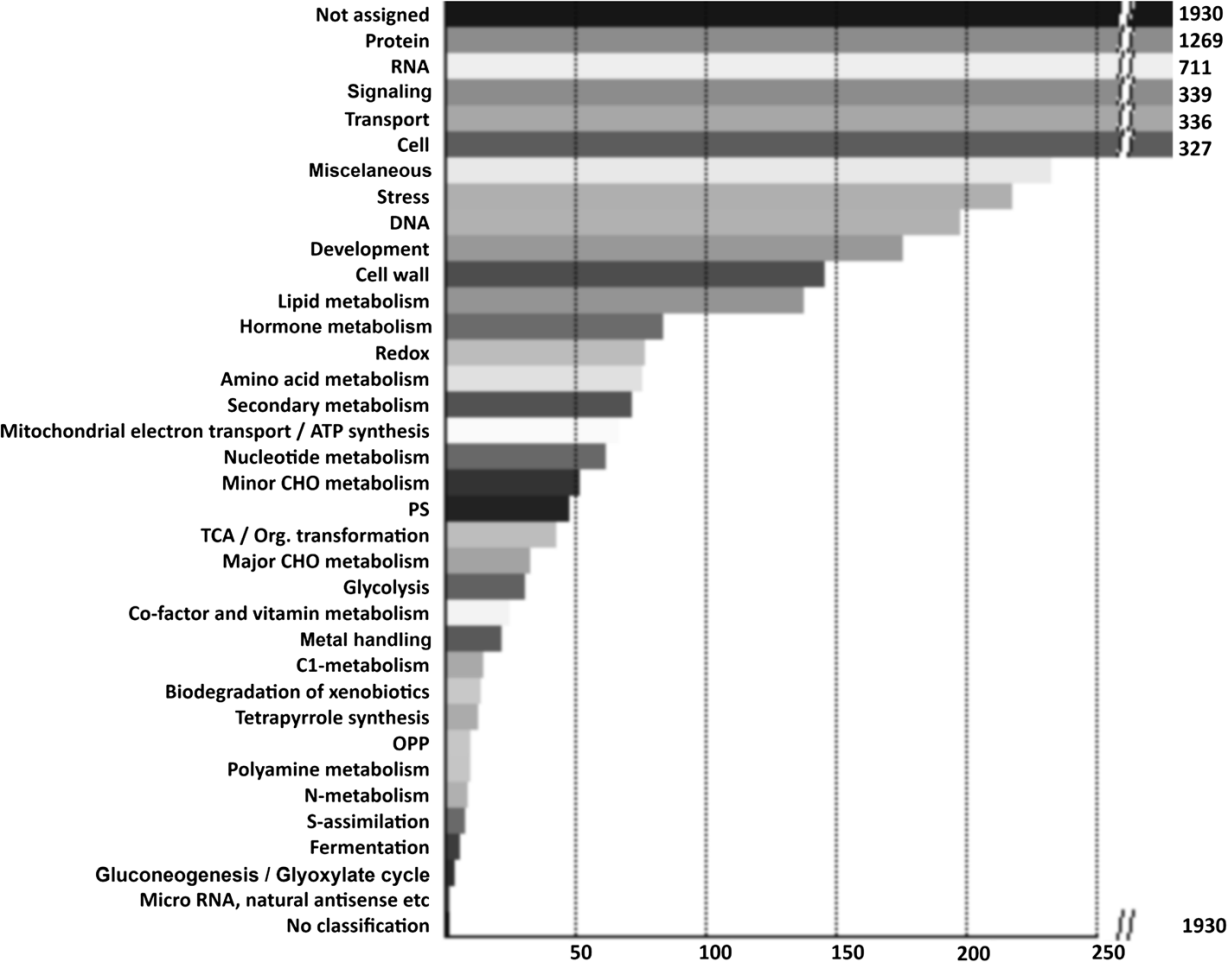
**Functional classification of wild-type Arabidopsis pollen tube transcriptome.** Analysis was performed with Classification SuperViewer Tool using MapMan source [13].

The transcriptome of wild type pollen tubes (6886 genes) was categorized into 34 functional groups according to the MapMan visualization software (Figure 1). Of the genes indicated as present, 28.7% had unknown functions, whereas the rest were primarily involved in protein synthesis (18.8%), RNA transcription and processing (10.6%), signaling (5.0%), transport (5.0%), and cell organization and cytoskeleton (4.9%). This functional distribution is consistent with the recruitment of cell resources for pollen tube growth, and is in agreement with other pollen and pollen tube microarray studies [4,5].

### *Arabidopsis agp6 agp11* differentially expressed genes

**Figure 2:**
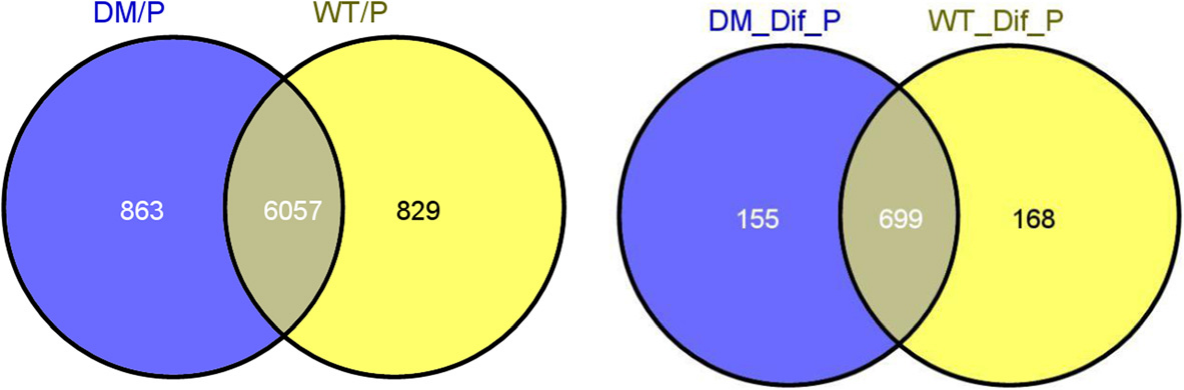
**Comparison of the wild-type and *agp6 agp11 *pollen tube transcriptomes.** Number of genes identified as being “Present” in wild-type pollen tubes (WT/P) and in *agp6 agp11* pollen tubes (DM/P) are shown on the left, and genes “Present” among the differentially expressed genes in wild-type pollen tubes (WT_Dif_P) and *agp6 agp11* pollen tubes (DM_Dif_P) are shown on the right. Among the whole set of 1022 differentially expressed genes, 155 were only present in the double mutant, and 168 genes were only present in the wild-type. The remaining pool of 699 genes contained both those which were up- and down-regulated.

The number of genes expressed in the *agp6 agp11* mutant pollen tubes was roughly equal to that of the wild type control although only 87.5% of expressed genes were common to both data sets (Figure 2). There are two gene populations of specific interest; 1) those present only in the double mutant and 2) those present only in the wild type pollen tubes. However, a statistical analysis of the significantly differentially expressed genes necessarily produced a rather different population of genes from that shown in Figure 2. Genes were considered to be differentially expressed from those they were compared with if the 90% lower confidence bound of the fold change between experiment and baseline was above 1.3, resulting in a median False Discovery Rate (FDR) of less than 5%. Observing such criterion a population of 1022 differentially expressed genes was obtained which accounts for 14.7% of the pollen tube transcriptome (Figure 2 and Additional file 1). These 1022 genes included 155 genes present only in *agp6 agp11*, 168 genes present only in wild type, and 699 genes expressed in both. About 60% of these 699 genes were up-regulated in *agp6 agp11*, with the remaining 40% being down-regulated. Overall the *agp6 agp11* mutation caused an up-regulation of over 500 genes in Arabidopsis pollen tubes.

### Validation of gene expression

RNA levels were independently verified for a collection of genes, either by real-time PCR (qPCR) or by conventional semi-quantitative RT-PCR. Genes were selected on the basis of expression differences between the two microarray data sets and of absolute signal values (Table 1). The relative expression levels for all genes tested were consistent with the results of the microarray experiments, thus confirming its reliability.

**Table 1:** Summary of confirmatory PCR assays

**Gene**	**AGI ID**	**LBFC**^**a**^	**FC - qPCR**^**b**^	**RT-PCR**^**c**^
Ubiquitin-conjugating enzyme 20	At1g50490	+2.44	+7.0	
Proline transporter 1	At2g39890	+2.02	+2.0	
Zinc finger (C3HC4-type RING finger) family protein	At5g60250	+1.96	+4.0	
Sucrose phosphate synthase 2 F	At5g11110	+1.70	+1.5	
Expansin B5	At3g60570	+1.50	+2.0	
CML42	At4g20780	+1.48	+4.5	
Plant cadmium resistance 11	At1g68610	-1.99	-2.0	
Cellulase 3	At1g71380	-1.98	-1.4	
Phosphoinositide 4-kinase gamma 4	At2g46500	-1.75	-2.2	
Heat shock protein 17.6A	At1g53540	-1.34	-2.2	
CAP (Pathogenesis-related protein, putative)	At2g19970	+23.34		+
C2 domain-containing protein	At3g57880	+16.93		+
F-box family protein	At1g65760	+11.8		+
Beta glucosidase 36	At1g51490	+4.95		+
CAP (allergen V5)	At2g19980	+4.79		+
Hypothetical protein	At2g22340	-141.79		-
Pentatricopeptide (PPR)	At5g28380	-44.55		-
Avirulence-responsive family protein	At4g09950	-25.77		-
DET3	At1g12840	-12.71		-

^a^ Lower bound of fold change (microarray experiment).

^b^*agp6 agp11* to wild-type fold change for qPCR experiments.

^c^ Genes up- or down-regulated in agp6 agp11, as assessed by semi-quantitative RT-PCR are indicated with a (+) or a (−) sign, respectively.

### Functional classification of differentially expressed genes

**Figure 3:**
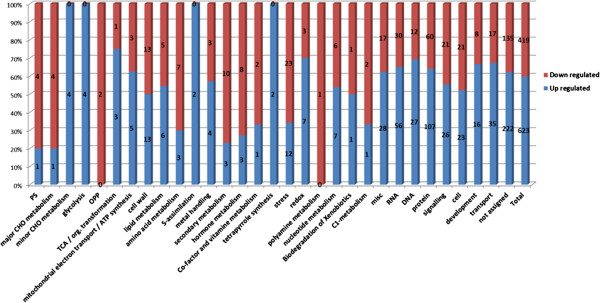
**Classification of differentially expressed genes according to MapMan software.** The proportion of genes, up- or down- regulated, within a given gene classification type is indicated and the actual number of genes this represents is shown.

According to the MapMan classification software, the functional classification of the entire double mutant transcriptome, does not substantially diverge from the functional classification of the wild type transcriptome (Figure 1). However, a significant number of genes are differentially expressed, i.e. they are either up- or down-regulated in response to the absence of two AGP gene products. The MapMan functional classification of these differentially expressed genes is shown in Figure 3.

The MapMan functional BINs “Protein”, “RNA”, “Transport”, “Signaling”, “Cell”, “DNA, and “Stress” are the groups in which the greatest number of genes was altered, and together account for almost half of the differentially expressed genes (46%).

A sub-cluster of the larger “Protein” cluster that contains genes with F-box motifs was particularly striking for its differential signal intensities and number of genes affected, with a representation factor of 1.8× relative to the Arabidopsis genome. Likewise, the sub-clusters “signaling.calcium”, and “stress.abiotic.heat” show enrichment factors of 1.5× and 2.1×, respectively, in the differentially expressed gene set, as compared to the whole genome. The sub-clusters “signaling.receptor.kinases” and “stress.biotic” were not enriched in the differentially expressed group of genes but were selected because of the high LBFC value of some of its members (Table 2).

**Table 2:** ***agp6 agp11*** differentially expressed genes among the MapMan BINs [29.5.11.4.3.2: protein. degradation. ubiquitin. E3. SCF. F-box], [30.2: signaling.receptor kinases], [30.3: signaling.calcium], [20.1:stress.biotic], and [20.2.1: stress.abiotic.heat]

**MapMan BIN**	**AGI locus**	**LBFC**^**a**^	**Gene annotation**^**b**^
29.5.11.4.3.2: protein. degradation. ubiquitin. E3. SCF. F-box			
Up-regulated			
	At1g65760	11.8	Protein of unknown function (DUF295)
	At2g27520	6.63	F-box and associated interaction domains-containing protein
	At5g38390	5.43	F-box/RNI-like superfamily protein
	At3g52030	3.92	F-box family protein with WD40/YVTN repeat domain
	At2g24080	3.52	Protein of unknown function (DUF295)
	At5g27750	3.19	F-box/FBD-like domains containing protein
	At3g49520	2.79	F-box and associated interaction domains-containing protein
	At1g70360	2.65	F-box family protein
	At3g17710	2.40	F-box and associated interaction domains-containing protein
	At2g17020	2.25	F-box/RNI-like superfamily protein
	At3g13820	2.08	F-box and associated interaction domains-containing protein
	At2g03580	1.99	F-box family protein-related
	At5g03000	1.75	Galactose oxidase/kelch repeat superfamily protein
	At1g06630	1.71	F-box/RNI-like superfamily protein
	At3g50080	1.61	VFB2, VIER F-box proteine 2
	At5g60610	1.55	F-box/RNI-like superfamily protein
	At3g18980	1.50	EIN2 targeting protein1 (ETP1)
	At3g59000	1.44	F-box/RNI-like superfamily protein
	At2g07120	1.38	F-box associated ubiquitination effector family protein
	At5g49610	1.36	F-box family protein
	At1g55590	1.35	RNI-like superfamily protein
	At1g23780	1.34	F-box family protein
Down-regulated			
	At5g43190	−7.32	Galactose oxidase/kelch repeat superfamily protein
	At1g68050	−4.00	“Flavin-binding, kelch repeat, F-box 1”
	At1g12490	−3.55	F-box associated ubiquitination effector family protein
	At1g55000	−1.99	Peptidoglycan-binding LysM domain-containing protein
	At1g78840	−1.96	F-box/RNI-like/FBD-like domains-containing protein
	At2g25490	−1.58	EIN3-binding F-box protein 1
	At4g04690	−1.52	F-box and associated interaction domains-containing protein
	At1g70590	−1.44	F-box family protein
30.2: signaling. receptor kinases			
Up-regulated			
	At4g23280	2.27	Cysteine-rich RLK (RECEPTOR-like protein kinase) 20
	At3g21970	2.22	Domain of unknown function (DUF26)
	At5g40380	1.33	Cysteine-rich RLK (RECEPTOR-like protein kinase) 42
	At4g20790	1.76	Leucine-rich repeat protein kinase family protein
	At3g24550	1.61	Proline extensin-like receptor kinase 1
	At4g28670	1.41	Protein kinase family protein with domain of unknown function (DUF26)
	At5g23170	1.35	Protein kinase superfamily protein
Down-regulated			
	At4g20530	−7.70	Receptor-like protein kinase-related
	At5g59650	−3.61	Leucine-rich repeat protein kinase family protein
	At4g02420	−2.87	Concanavalin A-like lectin protein kinase family protein
	At1g11280	−2.01	S-locus lectin protein kinase family protein
	At1g61380	−1.73	S-domain-1 29
30.3: signaling. calcium			
Up-regulated			
	At1g18890	2.66	Calcium-dependent protein kinase 1
	At5g19360	2.30	Calcium-dependent protein kinase 34
	At5g62390	1.57	BCL-2-associated athanogene 7
	At4g20780	1.48	Calmodulin-like 42 Calcium; sensor involved in trichome branching
	At1g05150	1.44	Calcium-binding tetratricopeptide family protein
	At5g42380	1.40	Calmodulin-like 37
	At1g51960	1.38	IQ-domain 27
	At5g47100	1.31	Calcineurin B-like protein 9
Down-regulated			
	At1g18530	−1.89	EF hand calcium-binding protein family
	At5g55990	−1.88	Calcineurin B-like protein 2
	At2g33990	−1.68	IQ-domain 9
	At1g62480	−1.54	Vacuolar calcium-binding protein-related
	At2g41410	−1.48	Calcium-binding EF-hand family protein
	At4g34150	−1.33	Calcium-dependent lipid-binding (CaLB domain) family protein
20.1: stress. biotic			
Up-regulated			
	At2g19970	23.34	CAP (Cysteine-rich secretory proteins, Antigen 5, and Pathogenesis-related 1 protein) superfamily protein
	At3g02840	2.56	ARM repeat superfamily protein
	At4g16920	2.52	Disease resistance protein (TIR-NBS-LRR class) family
	At1g32210	1.78	Defender against death (DAD family) protein
	At2g35520	1.77	Defender against death (DAD family) protein
Down-regulated			
	At4g09950	−25.77	P-loop containing nucleoside triphosphate hydrolases superfamily protein
	At3g44400	−6.01	Disease resistance protein (TIR-NBS-LRR class) family
	At4g16860	−4.48	Disease resistance protein (TIR-NBS-LRR class) family
	At1g75040	−2.63	Pathogenesis-related gene 5
	At3g18690	−1.88	MAP kinase substrate 1
	At1g14530	−1.82	TOM THREE HOMOLOG 1 (THH1); Protein of unknown function DUF1084
	At1g58170	−1.59	Disease resistance-responsive (dirigent-like protein) family protein
	At2g25240	−1.41	Serine protease inhibitor (SERPIN) family protein
20.2.1: stress. abiotic. heat			
Up-regulated			
	At1g28210	2.40	DNAJ heat shock family protein
	At3g22530	1.97	Unknown protein
	At1g72070	1.50	Chaperone DnaJ-domain superfamily protein
Down-regulated			
	At2g29500	−4.93	HSP20-like chaperones superfamily protein
	At3g46230	−1.92	Heat shock protein 17.4
	At5g12030	−1.84	Heat shock protein 17.6A
	At1g79920	−1.84	Heat shock protein 70 (Hsp 70) family protein
	At1g59860	−1.78	HSP20-like chaperones superfamily protein
	At5g22060	−1.65	DNAJ homologue 2
	At1g71000	−1.49	Chaperone DnaJ-domain superfamily protein
	At2g03020	−1.49	Heat shock protein HSP20/alpha crystallin family
	At4g13830	−1.43	DNAJ-like 20
	At5g02500	−1.41	Heat shock cognate protein 70-1
	At1g53540	−1.34	HSP20-like chaperones superfamily protein

^a^*agp6 agp11* to wild type lower bound of fold change.

^b^ TAIR annotations [14].

Another group of stress-related genes (CAP genes; Table 3) was also notable for their signal intensities and, in particular the large increase in the respective signal intensities of two of its members compared to wild type (Table 3).

**Table 3:** Summary of expression data for genes belonging to the CAP (Cysteine-rich secretory proteins, Antigen 5, and Pathogenesis-related 1 protein) superfamily protein

**AGI locus**	**agp6 agp11 signal**	**Wild type signal**	**LBFC**^**a**^	**Wild type call**^**b**^	**Pollen tube call**	**Pollen specificity**
At2g19970	299	7.5	+ 23.0	A	A	No
At2g19980	5622	887	+ 4.8	P	P	Yes
At3g19690	4785	4400	<1.3	P	P	Yes
At1g01310	5490	4100	<1.3	P	P	Yes
At4g25780	4578	4036	<1.3	P	P	Yes
At3g09590	1367	1395	<1.3	P	P	No
At5g02730	672	686	<1.3	P	P	No

^a^*agp6 agp11* to wild type lower bound of fold change.

^b^ A = absent; P = present.

**Figure 4:**
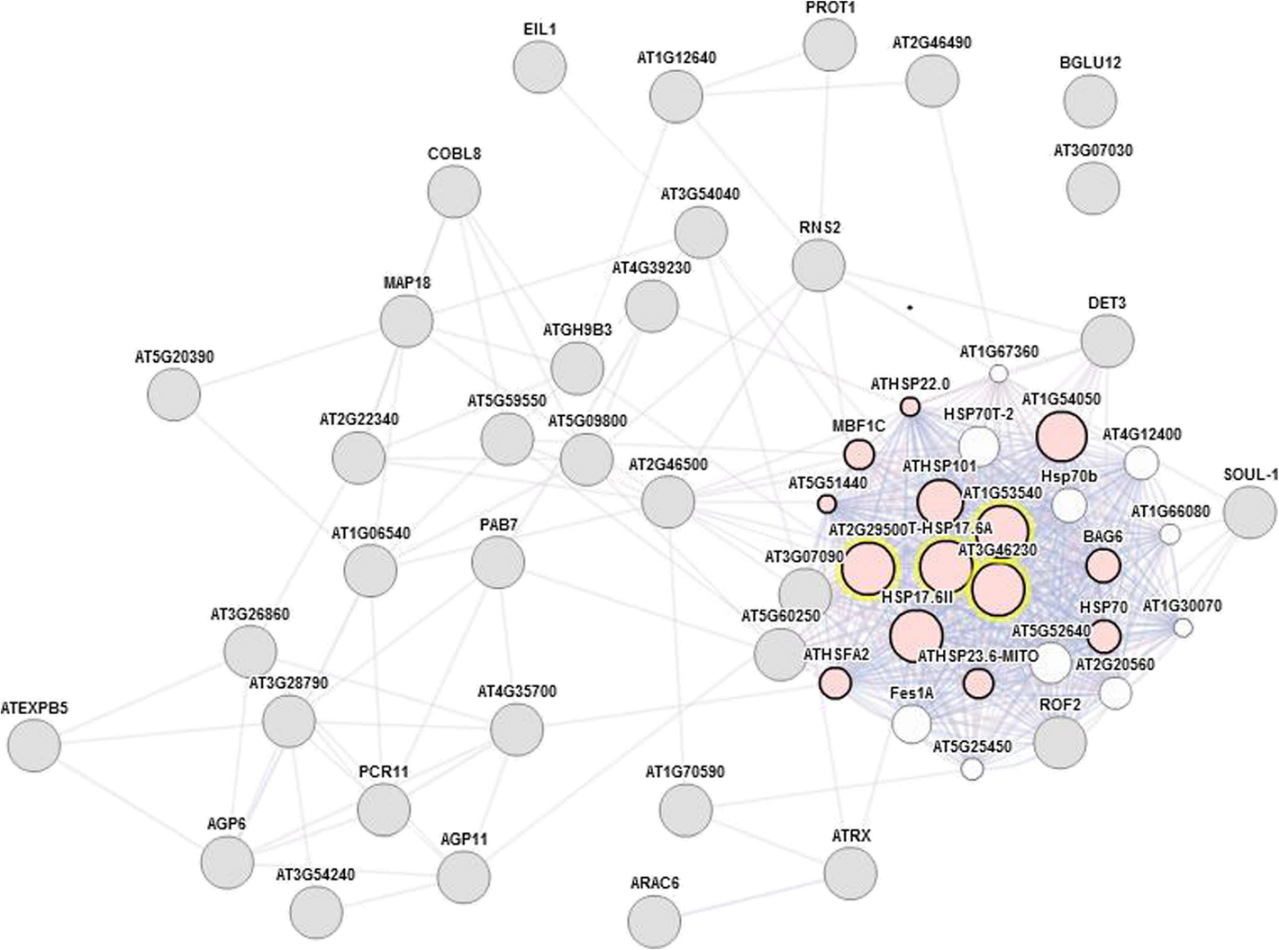
**Heat shock protein genes are differentially expressed in the *agp6 agp11 *pollen tubes.** The internet tool GeneMANIA was used to produce a gene association network. The input data was all the differentially expressed genes isolated from the 500 genes with highest signal intensities in the wild-type microarray data set. Some of those genes (At1g53540, HSP20-like chaperones superfamily protein; At3g46230, 17.4 kDa class I heat shock protein; At5g12030, heat shock protein 17.6A; At2g29500, HSP20-like chaperones superfamily protein) belong to a “response to heat” functional cluster (pink circles) [15].

To identify possible gene interactions and/or associations we used a Web interface (GeneMANIA) which is designed to analyze gene lists and prioritize genes for functional assays [15]. Extracting the differentially expressed genes from the list of 500 genes with the highest signal intensity, a gene network was produced that highlighted a “response to heat” gene cluster (Figure 4).

### AGP6 And AGP11 interactors detected by yeast-two hybrid assays

To experimentally detect putative interactors for AGP6 and AGP11 we used the core domains of these two proteins as baits to screen a normalized Arabidopsis expression library. A whole plant cDNA library was used as it was thought to be the best choice for identifying AGP interactors. The library was generated from pools of cDNAs obtained by mRNA extracted from several organs at different developmental stages including developing inflorescences, developing siliques, and mature flowers just before, and at, anthesis. The library was normalised in order to achieve a better representation of low abundant messengers.

For the AGP6 library mating plates, 111 colonies grew on selective media. Forty-eight among them were selected randomly for sequencing and they revealed 37 unique possible candidates. The ones that had an absent call in our array experiment as well as in other publicly available pollen and pollen tube arrays [5-7] were excluded, resulting in a set of 22 likely candidates for interaction (Table 4). For the AGP11 library mating plates only 11 colonies grew on selective media and all were sequenced. Those resulted in 5 likely candidates for interaction (Table 4).

**Table 4:** Interactors of AGP6 and AGP11 identified by Yeast two-Hybrid library screening assays

	**AGI locus**	**LBFC**^**a**^	**Gene description**^**b**^	**Expression in mature pollen**^**c**^	**Expression in pollen tube**^**c**^	**Subcellular localization**^**d**^	**Other**
AGP6	At1g62750	−1.66	Elongation factor G (SCO1)			Apoplast [16]	
	At1g70810	−1.02	Calcium-dependent lipid-binding (CaLB domain) family protein	[5-7]	[5,6]		
	At5g05010	−1.00	Clathrin adaptor complexes medium subunit family protein (MUG13.13)	[5-7]	[5,6]	Plasmodesmata [17], cytosol [18], plasma membrane [19]	Predicted GPI-attachment [20], predicted secretory pathway [21], predicted beta-barrel TM domain [22]
	At3g06650	−0.96	ATP-citrate lyase B-1	[7]	[6]		
	At1g32640	−0.95	MYC-related transcriptional activator (MYC2)		[5,6]		No predicted binding site (cacatg), but two similar sites (acacaag) in AGP6 promoter region [23]
	At1g67090	−0.93	Ribulose bisphosphate carboxylase small chain 1A (RBCS1A)	[5-7]	[5,6]	Apoplast [16]	Predicted secretory pathway [21]
	At5g55070	−0.91	Dihydrolipoamide succinyltransferase	[5], [6]	[5,6]	Mitochondrion [24]	
	At2g20580	−0.86	26S proteasome regulatory subunit N1 (RPN1A)	[6]	[6]	Cytosol [18], plasmodesmata [17]	One to five predicted alpha-helix TM domains [22], ubiquitin-binding subunit of proteasome complex [25]
	At3g61190	−0.85	BON1-associated protein 1 (BAP1)	[5], [7]	[5,6]	Plasma membrane [26]	
	At3g09440	−0.83	Heat shock protein 70-3	[5-7]	[5,6]	Cytosol [18], plasma membrane [27,28], cell wall [29], tonoplast [30], Golgi [31], apoplast [16]	Predicted secretory pathway [21]
	At5g21105	−0.77	L-ascorbate oxidase	[6]	[6]	Plasmodesmata [17], cell wall [32]	Predicted secretory pathway [21], predicted alpha-helix TM domains [22]
	At5g28540	−0.52	Luminal-binding protein 1 (BIP1)	[5-7]	[5,6]	Cell wall [29], cytosol [18], plasmodesmata [17], plasma membrane [27], ER [33], tonoplast [30], chloroplast [32]	Predicted secretory pathway [21], one to two predicted alpha-helix TM domains [22]
	At5g57560	−0.43	Xyloglucan endotransglucosylase/hydrolase 22 (TCH4, XTH22)		[6]	Cell wall [24]	Predicted secretory pathway [21], predicted alpha-helix TM domain and predicted beta-barrel TM domain [22]
	At5g15090	0.55	Mitochondrial outer membrane protein porin 2 (VDAC3)	[6]	[6]	Plasma membrane [19,28], cell wall [29]	Predicted beta-barrel TM domain [22]
	At1g16920	0.71	Ras-related small GTP-binding protein (RABA1b)	[7]			
	At3g18040	0.75	Mitogen-activated protein kinase 9 (MAPK9)	[5-7]	[5,6]		Predicted GPI-attachment [20], predicted alpha-helix TM domain [22]
	At4g05320	0.84	Polyubiquitin 10 (UBQ10)	[5-7]	[5,6]		
	At2g20990	0.87	Synaptotagmin A (SYTA)	[7]	[6]	Endosome [34], plasma membrane [19,28,35], ER [32]	Predicted secretory pathway [21], predicted alpha-helix TM domain [22]
	At1g26630	0.89	Eukaryotic translation initiation factor 5A-2 (FBR12)	[5-7]	[5,6]		Predicted GPI-attachment [20]
	At3g10740	0.92	Alpha-L-arabinofuranosidase 1 (ASD1)	[6], [7]	[6]	Apoplast [16], cell wall [36]	Predicted secretory pathway [21], one to two predicted alpha-helix TM domains [22]
	At5g11200	1.22	DEAD-box ATP-dependent RNA helicase 56	[6]	[5,6]	Cell wall [29], plasmodesmata [17]	
	At1g31150	2.50	Uncharacterized protein	[7]			Predicted secretory pathway [21], predicted alpha-helix TM domain [22]
AGP11	At3g47810	−1.43	MAG1 Homolog of yeast retromer subunit VPS29	[5-7]	[5,6]	Multivesicular body [37], endosome [38]	Mediator of protein targeting to vacuole [39]
	At1g42990	−0.97	bZIP transcription factor 60 (BZIP60)	[5-7]	[5,6]		Predicted GPI-attachment [20], predicted alpha-helix TM domain [22]
	At1g32640	−0.95	MYC-related transcriptional activator (MYC2)		[5,6]		Two predicted binding sites in AGP11 promotor region [23]
	At2g19070	−0.63	Spermidine hydroxycinnamoyl transferase (SHT)				Tapetum-specific [40], involved in exine formation [40,41]
	At2g30020	0.74	Putative protein phosphatase 2C 25	[5-7]	[5,6]	Nucleus [42,43], cytoplasm [43]	

^a^*agp6 agp11* to wild type lower bound of fold change.

^b^ TAIR gene annotations [14].

^c^ in published microarray experiments.

^d^ empirically determined in published references.

**Figure 5:**
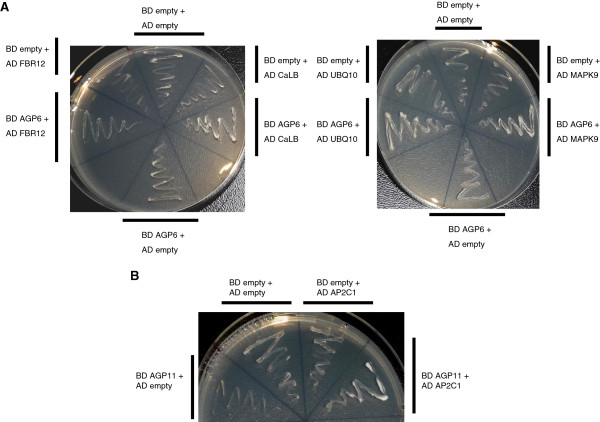
**Yeast two-hybrid experiments with candidate genes selected from the library screening.** YSD media -tryptophane, -leucine, -histidine 1 mM 3-Amino-1,2,4-triazole. Empty AD and BD vectors were used as negative controls. (**A**) Assays with* AGP6* protein core as bait and CaLB domain family protein (At1g70810), MAPK9 (At3g18040), UBQ10 (At4g05320) and FBR12 (At1g26630) as preys. (**B**) Assays with *AGP11* protein core as bait and AP2C1 (At2G30020) as prey.

Of the putative interactors uncovered, four were selected to confirm interaction with AGP6 and one with AGP11: CaLB domain family protein (At1g70810), MAPK9 (At3g18040), UBQ10 (At4g05320) and FBR12 (At1g26630), and AP2C1 (At2G30020), respectively. All co-transformed colonies with the AGP and each candidate grew on all selective media, while each negative control did not, thus confirming the interaction (Figure 5).

## Discussion

Sexual plant reproduction relies on the transfer of pollen to the stigma, where it hydrates and germinates into a pollen tube that extends through the pistil tissues to its target, the embryo sac. The *agp6 agp11* double null mutant, besides showing a pollen tube growth defect also shows an early “inside the anther” germination phenotype. These deficiencies, provide further evidence for the role of AGPs in cell wall deposition and growth, and potentially represent a defect in receiving, or responding to, germination cues.

AGPs are a class of molecules whose study has been particularly challenging. More than 90% of the molecular mass is carbohydrate (predominantly consisting of arabinose and galactose residues), and the role of the polypeptide chain, if any, is unknown. Moreover, how the sugar content and quality varies between different gene products, and whether different gene products are identically glycosylated is equally poorly characterised. A direct consequence of this is that techniques and approaches used to study proteins often do not apply to AGPs. The massive sugar component which surrounds the protein core is likely to have a biological role but its biological interactions and mechanism of action have so far eluded characterisation. Phenotypic differences between wild type and double mutant pollen tubes can only be attributed to the lack of both AGP6 and AGP11. It is impossible to determine whether the differential gene expression is due to a direct effect of the lack of AGPs on the expression of these genes, or whether the altered expression profile is a secondary and unspecific consequence of the reduction in growth rate. However, an expression-based study should improve our understanding of AGPs and their functionality by identifying genes that are altered in response to the lack of specific AGPs. This type of investigation is likely to be particularly informative when applied to a process known to involve AGPs, in a plant structure, the pollen tube, whose transcriptome is less than a third of the genome.

This study yielded a number of candidate genes that may directly or indirectly interact with AGPs and have a role in pollen tube growth. However, a comparative analysis of the present data with the expression profile of Arabidopsis culture cells subjected to Yariv phenylglycoside reagent, which specifically binds to and precipitates AGPs [44-48], was considered to be a valuable aid to the identification of genes which may be directly affected by the lack of AGP6 and AGP11.

### Microarray screen

The overall difference obtained for expressed genes was substantial, with 1022 differentially expressed genes in the double null mutant, compared with the wild type control. These 1022 differentially expressed genes could be further classified into three subgroups: 155 genes that were expressed “de novo” in the double mutant, 168 genes that were absent in the mutant but present in the control, and a group of 699 genes whose expression levels were found to be altered but that did not affect the present/absent status.

The gene clustering using MapMan software shows that the cluster “Protein” contains the largest fraction of differentially expressed genes. “Protein degradation” is undoubtedly the most represented group, in which the category of ubiquitin-mediated protein degradation is evident, with several of those genes belonging to the F-Box protein family. The F-box is a protein motif of approximately 50 amino acids that function as a site for protein-protein interaction [49]. Arabidopsis alone was reported to contain approximately 700 F-Box genes [50]. By comparison, only 20, 27, and 69 F-Box proteins are encoded by *Saccharomyces cerevisiae*, *Drosophila melanogaster*, and human genomes, respectively [51]. Such an apparent massive investment has also been found to be consistent with genetic studies linking specific plant F-Box proteins to numerous processes, including hormone perception and signaling, stress protection, chromatin remodeling, homeostasis, circadian rhythms, self-incompatibility, and defense against pathogens [52]. In Arabidopsis, recent genetic studies provided unequivocal evidence for the role of this protein degradation system during gametogenesis, which involved F-Box 17 [53] and a pair of ubiquitin-specific proteases (UBP3/UBP4) [54].

The analysis of the data obtained in the present work also shows calmodulins (CaMs), in the cluster of signaling by calcium, among the differentially expressed genes with highest LBFC score, namely At5g24880 and At4g20780 (CML42), the latter functioning in trichome branching and being strongly expressed in pollen as shown by CML42::GUS transgenic plants [55]. Calcium signals are known to play important roles in plant growth and development, including pollen tube growth, and CaMs are the most ubiquitous calcium sensors in eukaryotes. Popescu et al. [56] developed a protein microarray to comprehensively investigate Arabidopsis CaM/CaM-like interactions and identified new targets for these calcium sensors, one of which was an F-Box protein. The relationship between AGPs and calcium is unsurprising, as these proteins are involved in pollen tube cell wall growth, and calcium is known to regulate pollen tube growth and fertilization [57]. The data obtained may suggest an interaction of AGPs with calcium through calmodulin. This was also implied using an alternative approach to study AGP function which involved the use of (β-D-Glc)-3 Yariv reagent that specifically binds to, and presumably inactivates, a wide range of AGPs. Guan and Nothnagel [44] analyzed the gene expression profile of Arabidopsis culture cells subjected to Yariv phenylglycoside reagent for 1 and 10 h in a microarray study. Despite the specificities of the biological materials, several differentially expressed genes were found to be common between both experiments (Additional file 2). The values of LBFC used in the present work could not be used for direct comparison with the data of Guan and Nothnagel [44]. However, all values of fold change of *agp6 agp11* genes shown in Additional file 2, have values of LBFC above the threshold of 1.3, which was the criterion used in our analysis. Genes altered in Yariv-treated cells also include several heat-shock protein genes, which is a functional group also found to be affected in *agp6 agp11*.

Comparative pistil transcriptome analyses [58,59] identified a number of genes encoding proteins potentially involved in stress and defense responses. This is particularly interesting as there is evidence that the molecules involved in pollination and stress/defense responses may be evolutionary and functionally related. Many authors have proposed that certain self-incompatibility mechanisms may have arisen through the modification of pre-existing pathogen defense mechanisms [60].

The analysis of the stress cluster of the present data shows that the genes associated with biotic and abiotic types of stress, namely PR and heat shock protein genes, had their expression levels significantly shifted. In this cluster, genes such as At2g19970 and At2g19980 code for proteins of the CAP (cysteine-rich, antigen 5 and pathogen-related 1 protein) superfamily. Members of the CAP superfamily are widespread in living organisms, [61], and have been intensely studied in mammalian fertilization mechanisms. They are most often secreted proteins and are involved in several processes including cell adhesion during fertilization. The plant pathogenesis proteins of the PR-1 family, which are synthesized during pathogen infection or other stress-related responses, belong to the CAP superfamily but the precise functions of these proteins are still unresolved. In a recent study [62] it was suggested that the glioma pathogenesis-related 1 (GLIPR1L1), along with other members of the CAP superfamily and several other proteins, are involved in the binding of sperm to the oocyte complex. Also, in a work of microarrays of rice embryo sac cells [63], it was revealed that allergen V5/Tpx-1-related proteins, which are members of the CAP superfamily, are abundant in the rice synergid cell. Collectively these findings strengthen the possible role of CAP domain-containing proteins in cellular adhesion and fertilization.

The high level of expression of 7 members of the CAP superfamily in Arabidopsis pollen tube is relevant given their predicted signaling function. Moreover, knowing that these proteins are most likely secreted and the fact that one of its members (At2g19970) is only present in the *agp6 agp11* double mutant, may indicate some type of relationship between AGPs and CAP processing.

Application of a different bioinformatic tool, GeneMania, a co-expression network was obtained which highlighted a stress functional gene cluster. Surprisingly, some of the genes correlated with those whose expression was found to be altered by treatment with Yariv reagent for 10 h (At2g29500, At3g46230, At1g53540 and At5g12030) [44]. Guan and Nothnagel [44] compared the genes that were induced by Yariv treatments with genes whose expression had been previously been shown to be induced by other conditions and concluded that the gene expression profile induced by Yariv treatment was similar to that of wound response. Knowing that pollen tube growth and discharge and the defense against fungal attack are alike in many respects, and that both pollen tubes and fungi exploit similar receptor proteins [64,65] we propose a role for AGPs in this process where, apparently, pollen tube and fungal hyphae activate plant cell responses.

### Yeast two-hybrid experiments

To identify possible AGP6 and/or AGP11 interactors, yeast two-hybrid experiments were performed. These experiments revealed some interesting partners involved in the proteosome-independent roles of ubiquitination in signaling and endocytosis.

Besides the well-known role in proteosome degradation, ubiquitin conjugation is also involved in down-regulation of membrane receptors, transporters and channels. Ubiquitination of plasma membrane proteins leads to their endocytosis into the multivesicular endosome and most members of the ubiquitin ligase family responsible for trafficking of diverse proteins carry an N-terminal calcium-dependent lipid/protein C2 domain that specifically binds phosphoinositides in yeast [66,67].

Endosomes are primarily intracellular sorting organelles, and receive proteins and lipids from both the biosynthetic and the endocytic pathways. Plasma membrane proteins that are internalized by endocytosis are either recycled back to the plasma membrane or sorted for degradation, which is achieved by intermediate/late endosomes, also called multivesicular bodies (MVBs) [68,69].

It is known that pollen tubes grow by rapid tip localized exocytosis, most probably coordinated with an also tip localized endocytosis. Using Arabidopsis and Nicotiana pollen tubes as models, Zhao et al. [70] showed that phosphatidylinositol-4-phosphate 5-kinase 6 (PIP5K6) regulates clathrin-dependent endocytosis in pollen tubes.

The internalization and secretion of arabinogalactan-rich glycoproteins through MVBs has been described but only microscopically [71,72]. The data obtained in the present study suggests a physiological interaction of AGPs with members of the endosomal system, and that AGP6 and AGP11, two specific and highly expressed proteins in Arabidopsis pollen and pollen tubes may be involved in this signaling pathway that sends and recycles proteins to the extracellular matrix. In the yeast two-hybrid screening assays, clathrin adaptor complexes were present such as MUG13.13 and MAPK9, SYTA (a transmembrane protein involved in membrane trafficking), RABA1b (a small GTP binding protein also involved in intracellular protein trafficking) and MAG1 (a homolog of the yeast retromer subunit VPS29). These results give further support for the clathrin machinery of receptor internalization in pollen tubes.

Although AGPs are presumably acting extracellularly, they are targeted to the outer leaflet of the plasma membrane through vesicles, which originate in the ER and Golgi network. During the trafficking into the extracellular matrix it is probable that AGPs interact with cytosolic proteins such as bZIP60 or FBR/eIF5A, two regulatory proteins that were identified in the yeast two-hybrid experiments.

With endocytosis established as an essential plant cell function [69] and AGP6 and AGP11 supposedly involved in this process, we looked further into the microarray data. Three Rab GTPases known as specific molecular markers of plant endosomes, Ara6 (At3g54840), Ara7 (At4g19640) and Rha1 (At5g45710, also a member of the heat stress transcription factor family) are all down-regulated in *agp6 agp11.* RabA2 (At3g46830) and RabA3 GTPases define a trans-Golgi endosomal membrane that overlays with VHA-a1 (At2g28520) one of the isoforms of a membrane integral V-ATPase specifically localized to the TGN [73]. Another subunit of V-ATPases, DET3, is one of the most down-regulated genes in the present study and known to be involved in plant growth and development [74].

A C2 domain is a protein structural domain involved in targeting proteins to cell membranes. C2 domains are modular lipid-binding domains found in a variety of proteins with functions that include vesicular transport, GTPase regulation, lipid modification, protein phosphorylation, and ubiquitylation [75]. The most extensively studied proteins included in the last group are the synaptotagmins (SYT). There are reports describing the involvement of C2-domain proteins in plant responses to abiotic and biotic stresses. Yang et al. [26,76] showed that the Arabidopsis C2-domain protein, BAP1, acts as a negative regulator of programmed cell death induced by biotic stimuli. This gene is differentially expressed in the present microarray experiment and in the Yariv microarray experiment described by Guan and Nothnagel [44]. The third most up-regulated gene in the present data set is a calcium-dependent lipid binding protein, CaLB (At3g57880), a C2 protein which is also up-regulated in the Yariv array. It seems relevant to highlight that the C2 calcium-dependent membrane targeting protein At1g70810 was identified as an AGP6 interactor in the yeast-two hybrid assay. Furthermore, in a yeast two-hybrid assay performed on Nicotiana alata [77] to identify pollen proteins potentially involved in deciphering chemical signals provided by the pistil, two pistil-specific AGPs from the transmitting tract were used to hybridize against a pollen cDNA library. The authors found three pollen proteins that interacted with those pistil AGPs, one of which was a C2 domain-containing protein (NaPCCP).

Analysis of the present results further identified a possible involvement of AGP6 and AGP11 in membrane trafficking, and that a plant retromer may be present in pollen tubes where the movement of vesicles is very important. A retromer is a multiprotein complex that is strongly conserved in eukaryotes. It is involved in the recycling of transmembrane receptors, which mediate the transport of vacuolar/lysosomal hydrolases [78]. In yeast, retromers consist of a large subunit with three vacuolar protein sorting (VPS) proteins, VPS35p, VPS29p and VPS26p. Homologs of these proteins are present in mammals and in plants. Recently, Oliviusson et al. [37] showed that the three VPS proteins, namely VPS35 (At3g51310), VPS29 (At3g47810), and VPS26 (At5g53530) of the plant retromer complex are localized to multivesicular bodies (MVBs) in tobacco BY2 cells. MAG1, a homolog of VPS29, is differentially expressed in the present array, as is VPS26. Besides, VPS29 is also differentially expressed in the Yariv microarray data and MAG1 is one of the AGP11 interactors in the yeast-two hybrid experiment. Recent data strongly suggest that the retromer complex has conserved a function in mediating retrograde trafficking [38] and that VPS29 is involved in the cycling of certain PM proteins and required for the establishment of cell polarity during organ initiation in plants [66].

**Figure 6:**
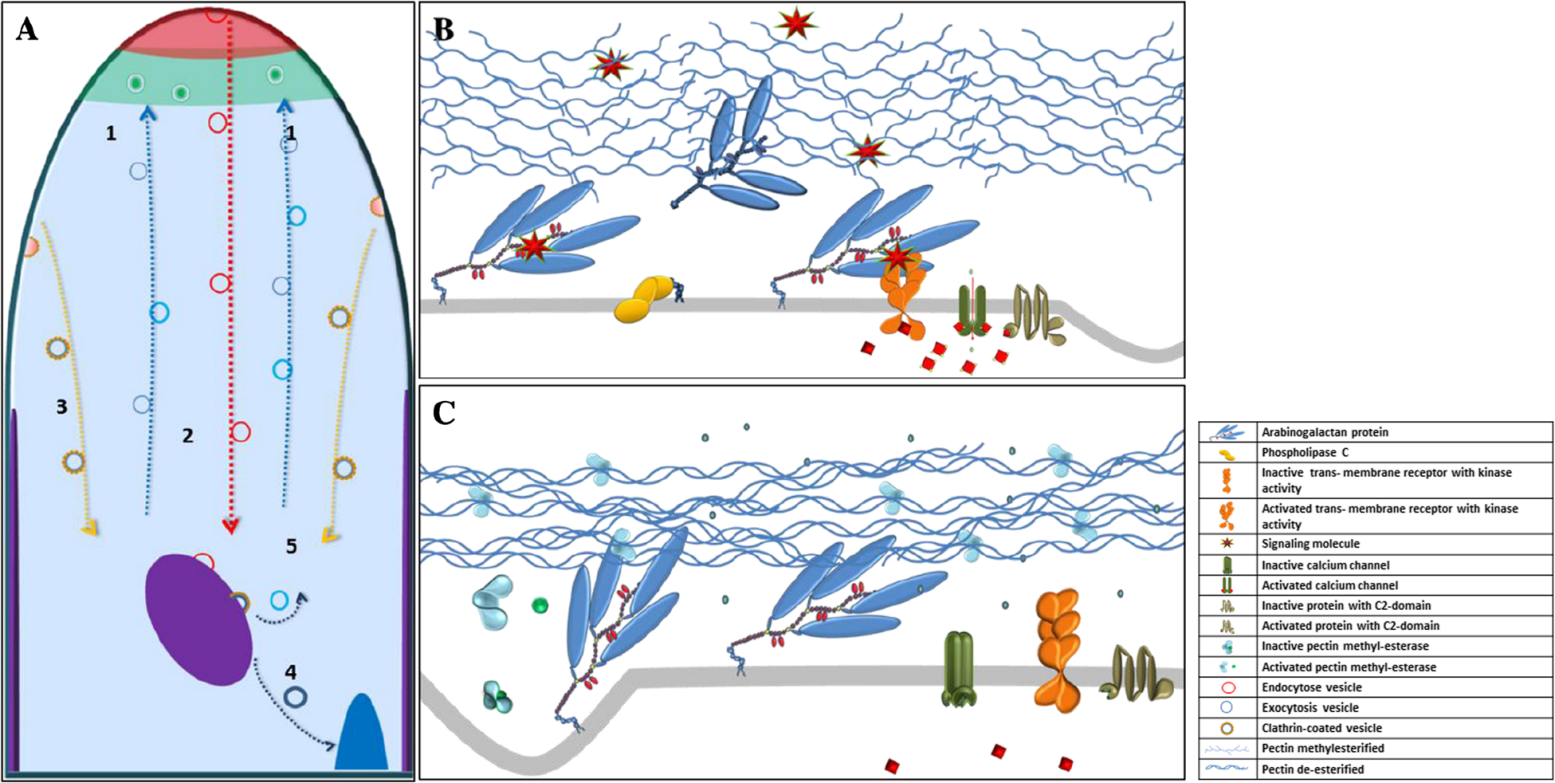
**A model for the role of AGPs in pollen tube growth.** (**A**) Accepted model for pollen tube growth [70,79], illustrating vesicle exocytosis (1), smooth endocytosis (2), clathrin vesicle endocytosis (3), the pathway into degradation (4) and the recycling pathway (5). The apical dome (red), the exocytosis region (green), the multivesicular body (purple) and the vacuole (blue) are shown. (**B**) Proposed model for AGP signaling role in the pollen tube apical dome. AGPs act as receptors for extracellular signals and interact with transmembrane proteins, possibly receptor kinases or C2 domain-containing proteins. These interactions lead to opening of calcium channels, triggering various intracellular events. During pollen tube growth, AGPs are recycled by endocytosis, and either reused or sent for degradation, through multivesicular bodies. (**C**) Proposed model for the sub-apical zone. The hardening of cell wall pectins resulting from complexing with calcium hinders AGP contact with putative signal molecules, rendering AGPs inactive.

A working model for the role of AGPs in pollen tube development which integrates the data obtained in this work is proposed (Figure 6).

## Conclusions

It is known that plant cells recycle pectins and AGPs [80]. Pectin cross-linking calcium may be removed and pectins recycled, maintaining the loosened walls essential for growth. AGPs can also be recycled, to maintain their concentration level at the pollen tube apex necessary for them to perform a signaling role.

All the data obtained in the present work emphasizes the remodeling of the plasma membrane via endocytosis. Endosomal trafficking pathways are emerging as fundamental regulators of the wall physiology, involved in multiple signaling pathways and developmental processes. The interaction of AGP6 and AGP11 with members of the pollen tube endosome machinery provides strong evidence for the recycling of these cell wall proteins (Figure 6).

It is our understanding that as we continue to unravel the signal transduction processes involved in intercellular communication during pollen/pistil interaction, we will discover more about the function of AGPs.

## Methods

### Plant material and growth conditions

A Ds-tagged *agp6 agp11* homozygous double null mutant line [11] obtained from crossing RIKEN lines Ds54-4754-1 and Ds11-4025-1 (RIKEN GSC *Arabidopsis* Ds transposon tag line collection, [81]), was used for pollen harvest and plants from the ecotype Nossen (No-0 Line N3081 NASC) were used as control. All plants were germinated and grown in half strength Murashige and Skoog (MS) medium complemented with 0.7% agar. Plantlets were then transferred to soil and kept in a growth chamber at 22°C under long days (16 h light/8 h dark), irradiance of 130 μmol m^-2^ s^-1^ and 60% relative humidity.

### Pollen isolation and pollen tube culture

Pollen was collected from newly opened flowers and germinated according to Dardelle et al. [82]. Forty open flowers of Arabidopsis were introduced in each 1.5 mL microtube, and gently vortexed with 1 mL of pollen growth medium (5 mM KCl, 5 mM CaCl_2_, 1 mM MgSO_4_, 0.01% H_3_BO_3_, 10% Sucrose, pH 7.4) for 5 min. The flowers were then removed with forceps, the microtubes centrifuged for 3 min at 3000 rpm and the supernatant discarded. The pelleted pollen destined for pollen tube culture was combined in groups of three and resuspended in 1.25 mL of fresh pollen growth medium. The preparation was then transferred to 20 mm diameter flat bottom flasks and incubated at 22°C in the dark for 8 h. The germinated pollen tubes were then washed through a 60 μm mesh nylon net filters (Millipore NY6004700) with growth medium in order to discard ungerminated pollen grains. Pollen tubes from approximately 1600 flowers were used to obtain each independent replicate for *agp6 agp11* and for wild type.

### RNA isolation, target synthesis and hybridization to affymetrix GeneChips

Total RNA was extracted using the RNeasy Mini Kit (Qiagen, Hilden, Germany). Concentration and purity was determined by spectrophotometry and integrity was confirmed using an Agilent 2100 Bioanalyzer with a RNA 6000 Nano Assay (Agilent Technologies, Palo Alto, CA).

RNA was processed for use on Affymetrix (Santa Clara, CA, USA) GeneChip Arabidopsis ATH1 Genome Arrays, according to the manufacturer’s GeneChip 3′ IVT Express kit user manual. Briefly, 100 ng of total RNA containing spiked in Poly-A RNA controls was used in a reverse transcription reaction (GeneChip 3′ IVT Express Kit; Affymetrix) to generate first-strand cDNA. After second-strand synthesis, double-stranded cDNA was used in a 16 h *in vitro* transcription (IVT) reaction to generate aRNA (GeneChip 3′ IVT Express Kit; Affymetrix). Size distribution of the aRNA and fragmented aRNA, respectively, was assessed using an Agilent 2100 Bioanalyzer with a RNA 6000 Nano Assay.

15 μg of fragmented aRNA was used in a 300 μl hybridization cocktail containing added hybridization controls. 200 μl of mixture was hybridized on arrays for 16 h at 45°C. Standard post hybridization wash and double-stain protocols (FS450_0004; GeneChip HWS kit, Affymetrix) were used on an Affymetrix GeneChip Fluidics Station 450. Arrays were scanned on an Affymetrix GeneChip scanner 3000 7 G.

### GeneChip data analysis

Scanned arrays were analyzed first with Affymetrix Expression Console software to obtain Absent/Present calls and to assure that all quality parameters were in the recommended range. Subsequent analysis was carried out with DNA-Chip Analyzer (dChip) 2010 (http://www.dchip.org, Cheng Li Lab, Harvard). The 6 arrays were normalized to a baseline array with median CEL intensity by applying an Invariant Set Normalization Method [83]. Normalized CEL intensities of the arrays were used to obtain model-based gene expression indices based on a PM (Perfect Match)-only model [84]. Replicate data (triplicates) for the conditions were weighted gene-wise by using inverse squared standard error as weights. Genes compared were considered to be differentially expressed if the 90% lower confidence bound of the fold change between experiment and baseline was above 1.3. The lower confidence bound criterion means that we can be 90% confident that the fold change is a value between the lower confidence bound and a variable upper confidence bound. Li and Wong [83,84] have shown that the lower confidence bound (LBFC) is a conservative estimate of the fold change and therefore more reliable as a ranking statistic for changes in gene expression.

### Quantitative real-time PCR (qPCR)

Samples from the same RNA preparations used in the microarray experiment were reverse transcribed using Promega Reverse Transcription System and poly(dT)_12–18_ to prime the reactions. cDNA was amplified using the iQ™ SYBR® Green Supermix on the iQ™5 Real-Time PCR Detection System (Biorad).

All qPCR reactions were run in duplicates. Thermocycle settings were as follows: Initial denaturation of 3 min at 95°C, followed by forty cycles, each consisting of 10 s at 95°C, 30 s at 56°C and 30 s at 72°C. After each run, a dissociation curve was acquired to check for amplification specificity by heating the samples from 60 to 95°C.

Serial dilutions of both *agp6 agp11* and wild type pollen tube cDNA were used to determine the efficiency curve of each primer pair. The primers used are listed in Table 5. UBQ10 was used as internal reference gene. At the end of the PCR cycles, the data was analyzed with the iQ5 2.0, Standard Edition Optical System Software v2.0.148.060623 (Biorad), using the Livak calculation method [85].

**Table 5:** Oligonucleotide primer sequences used in qPCR and in reverse-transcription PCR assays

**AGI ID**	**primer ID**	**Forward primer**	**Reverse primer**
At4g05320	UBQ10	gctccgacaccattgacaac	acgcaggaccaagtgaagag
At1g71380	ATCEL3	tctccaagtcattgctcttcttcc	cgttgtctcctgcgtcatagtac
At3g60570	ATEXPB5	gcaaacggtgatgggaacttcg	ggacacggcggaggtaagc
At5g11110	ATSPS2F	tggtggtgttcgtgggagattcag	ttagcctcggtgatgttgggactg
At1g53540	HSP17.6C-CI	ggcaaacgcacccgctatg	ttcacctcttccttcctcagtcc
AT2g46500	AT2g46500	agacggctcaaacgctcagaatc	cggatagggcttcgaggaatgc
At2g39890	PROT1	atggcgagaggcgggtac	gtggttggctagaatgaatgtgag
At1g50490	UBC20	agatcctccggcgtctaatgg	tgcttcctgttattgtccctttcc
At5g60250	AT5g60250	cggttacagatggaggaggcactc	aggacaccacacgtagccacaatc
At1g68610	At1g68610	ctctaacgaccaaccaagccaag	acatcgctccgctcacacc
At4g27960	UBC9	gtttcaccaccctttcttc	aaatcccacgatcaaattcc
At2g19970	At2g19970	gagacattctgatggaccttacg	tccgagacttaacgattgattgg
At1g65760	At1g65760	cgatgattcctacattagcagac	aagacgcaacgggtaacg
At2g22340	At2g22340	gcgacggtgggatttcaggaactg	gatgggagcggcaacggatgtg
At5g28380	At5g28380	aagcacactgcgaccaaggc	cagcggctcatggatctcactac
At4g09950	At4g09950	ggaggatgtgaaggagcaattagc	tcttgttgagttcggtgcgtaac
At1g12840	DET3	cggcgttcttggcatgtgtc	agcaaggttgatagtgaaggagac
At1g51490	BGLU36	gccgtactctctcgctgtcaaag	atgagccagaagttcgtgatgtcc
At3g57880	C2 domain-containing protein	caggatgaggtatrgacaggttgag	gcggcaatcaagcagaacaag
At4g20780	CML42	cccaagcctaaacgcacttcg	acggtggatttgagatcggagag
At2g19970	CAP (Antigen 5)	gagacattctgatggaccttacg	tccgagacttaacgattgattgg
At2g19980	CAP (allergen V5)	gcacagaggtacgctaacg	ggtggcataattgtaataaggc

### Semi quantitative reverse transcription (RT)-PCR

cDNA samples used in qPCR experiments were also used in the RT-PCR assays. RT-PCR reactions were done on 8 genes, and two reference genes, UBC9 and UBQ10 (Table 5). PCR reactions were set up with DreamTaq DNA Polymerase (Fermentas). Each PCR reaction was initiated with a 5 min denaturation at 95°C followed cycles of 30 s at 95°C, 30 s at 56°C (for all primer pairs) and 30 s at 72°C. Samples were taken after 25, 30 and 35 cycles. cDNA quantities were normalized for each sample using the two reference genes. Band intensity was assessed using Kodak DC120 Gel Electrophoresis Analysis System.

### Yeast Two-hybrid library screening assays

The predicted peptide cores of AGP6 and AGP11, followed by a stop codon, were individually cloned into the pGBTKT7 vector (Clontech, Palo Alto, CA), modified at the XmaI site to include a Gateway cassette. The bait constructs were introduced in the α-Y187 strain [86]. YPAD, SD, and appropriate dropout media have been described previously [87].

The bait strains were mated with a normalized *Arabidopsis thaliana* total plant cDNA library cloned in pGADrec (Sommer and Masiero, unpublished data) and introduced in the α-AH109 yeast strain (Clontech).

Colonies that grew on all selective media (−Trp-Leu-Adenine-His and supplemented with 15 mM 3-Amino-1,2,4-triazole) were further characterized. The pGADrec plasmids were rescued and cDNA insert was amplified by PCR and sequenced.

### Yeast Two-hybrid interaction assays

cDNA of each candidate gene was used to perform confirmation of the interaction. RNA was extracted from Arabidopsis inflorescences containing flowers in several stages of development using the RNeasy Mini Kit (Qiagen). cDNA was synthesized with the RevertAid First Strand cDNA Synthesis Kit (Thermo Scientific, Waltham, MA, USA). Full-length cDNA of these genes was amplified by PCR (Table 6) with Phusion High-Fidelity DNA Polymerase (New England Biolabs, Ipswich, MA, USA) and purified from agarose gel using the GeneJET Gel Extraction Kit (Thermo Scientific). In case of alternative splicing forms, the splice selected was the one corresponding to the mature form according to TAIR. The cDNA sequence of each gene was individually cloned into the pGADT7 vector (Clontech).

**Table 6:** Oligonucleotide primer sequences used in the DNA constructions for the Y2H assays

**AGI ID**	**primer ID**	**Forward primer**	**Reverse primer**
At5g14380	AGP6	ggggacaagtttgtacaaaaaagcaggctcggccgacgctccctcagcttctcc	ggggaccactttgtacaagaaagctgggtcctaactcttgggtgactctgcagtgg
At3g01700	AGP11	ggggacaagtttgtacaaaaaagcaggctcggccgatgcaccttcagctgcacc	ggggaccactttgtacaagaaagctgggtcctaacttttgggtgactcggcggc
At1g26630	FBR12	ggggacaagtttgtacaaaaaagcaggctcgaccttcctcttcccctcc	ggggaccactttgtacaagaaagctgggtcctagtgcaaatagcaatgaatatc
At4g05320	UBQ10	ggggacaagtttgtacaaaaaagcaggctcgaaatcttaaaaactttctctc	ggggaccactttgtacaagaaagctgggtcctaaaacaaaagaagcacagataat
At3g18040	MAPK9	ggggacaagtttgtacaaaaaagcaggctcggcgaaaagtttctcccttg	ggggaccactttgtacaagaaagctgggtcctatgaagaaaacacacactttaac
At1g70810	CaLB domain family protein	ggggacaagtttgtacaaaaaagcaggctcgaaaagagtcagagccgc	ggggaccactttgtacaagaaagctgggtcctaaaaccaaaacgatttagg
At2g30020	AP2C1	ggggacaagtttgtacaaaaaagcaggctcgcgaaatcgaaatcaaaatatag	ggggaccactttgtacaagaaagctgggtcctaaatttcggccaatgctcg

Each bait/prey pair was introduced in the α-AH109 yeast strain (Clontech), and as a control for autoactivation false-positives, each bait was also co-transformed into the yeast strain with the empty AD vector, and each prey was co-transformed with the empty BD vector. Bait/prey pair colonies that grew on all selective media (−Trp-Leu-Adenine-His and supplemented with increasing concentrations of 1 mM to 2.5 mM 3-Amino-1,2,4-triazole) were considered positive for interaction.

## Authors’ contributions

MC carried out the molecular genetic studies; MSN carried out the yeast-two-hybrid assays; SM designed the yeast-two-hybrid assays; JB carried out the microarray experiment; MIA participated in the data analysis; LGP participated in the design of the study and helped to draft the manuscript. SC, PI of the project conceived the study and its coordination and drafted the manuscript. All authors read and approved the final manuscript.

## Supplementary Material

Additional file 1 ***agp6 agp11* mutant microarray data.** Click here for file

Additional file 2 Differentially expressed genes common to agp6 agp11 mutant and treatment with Yariv phenylglycoside reagent for 1 h and 10 h. Click here for file

## References

[B1] Campanoni P Blatt MR Membrane trafficking and polar growth in root hairs and pollen tubes J Exp Bot 2007 58 1 65 74 16873451 10.1093/jxb/erl059

[B2] Becker JD Boavida LC Carneiro J Haury M Feijó JA Transcriptional profiling of Arabidopsis tissues reveals the unique characteristics of the pollen transcriptome Plant Physiol 2003 133 713 725 10.1104/pp.103.028241 14500793 PMC219046

[B3] Honys D Twell D Comparative analysis of the Arabidopsis pollen transcriptome Plant Physiol 2003 132 2 640 652 10.1104/pp.103.020925 12805594 PMC167004

[B4] Pina C Pinto F Feijó JA Becker JD Gene family analysis of the Arabidopsis pollen transcriptome reveals biological implications for cell growth, division control, and gene expression regulation Plant Physiol 2005 138 2 744 756 10.1104/pp.104.057935 15908605 PMC1150393

[B5] Wang Y Zhang WZ Song LF Zou JJ Su Z Wu WH Transcriptome analyses show changes in gene expression to accompany pollen germination and tube growth in Arabidopsis Plant Physiol 2008 148 3 1201 1211 10.1104/pp.108.126375 18775970 PMC2577266

[B6] Qin Y Leydon AR Manziello A Pandey R Mount D Denic S Vasic B Johnson MA Palanivelu R Penetration of the stigma and style elicits a novel transcriptome in pollen tubes, pointing to genes critical for growth in a pistil PLoS Genet 2009 5 8 e1000621 10.1371/journal.pgen.1000621 19714218 PMC2726614

[B7] Honys D Twell D Transcriptome analysis of haploid male gametophyte development in Arabidopsis Genome Biol 2004 5 11 R85 10.1186/gb-2004-5-11-r85 15535861 PMC545776

[B8] Borner GHH Sherrier DJ Weimar T Michaelson LV Hawkins ND MacAskill A Napier JA Beale MH Lilley KS Dupree P Analysis of detergent-resistant membranes in Arabidopsis. Evidence for plasma membrane lipid rafts Plant Physiol 2005 137 104 116 10.1104/pp.104.053041 15618420 PMC548842

[B9] Yariv J Lis H Katchalski E Precipitation of arabic acid and some seed polysaccharides by glycosylphenylazo dyes Biochem J 1967 105 1 1C 2C 6069833 10.1042/bj1050001c PMC1198314

[B10] Ellis M Egelund J Schultz CJ Bacic A Arabinogalactan-proteins: key regulators at the cell surface? Plant Physiol 2010 153 403 419 10.1104/pp.110.156000 20388666 PMC2879789

[B11] Coimbra S Costa M Jones B Mendes MA Pereira LG Pollen grain development is compromised in Arabidopsis agp6 agp11 null mutants J Exp Bot 2009 60 11 3133 3142 10.1093/jxb/erp148 19433479 PMC2718217

[B12] Coimbra S Costa M Mendes MA Pereira AM Pinto J Pereira LG Early germination of Arabidopsis pollen in a double null mutant for the arabinogalactan protein genes AGP6 and AGP11 Sex Plant Reprod 2010 23 3 199 205 10.1007/s00497-010-0136-x 20162305

[B13] Provart N Zhu T A browser-based functional classification superviewer for arabidopsis genomics Currents Comput Mol Biol 2003 271 272

[B14] Berardini TZ Mundodi S Reiser L Huala E Garcia-Hernandez M Zhang P Mueller LA Yoon J Doyle A Lander G Moseyko N Yoo D Xu I Zoeckler B Montoya M Miller N Weems D Rhee SY Functional annotation of the Arabidopsis genome using controlled vocabularies Plant Physiol 2004 135 2 745 755 10.1104/pp.104.040071 15173566 PMC514112

[B15] Warde-Farley D Donaldson SL Comes O Zuberi K Badrawi R Chao P Franz M Grouios C Kazi F Lopes CT Maitland A Mostafavi S Montojo J Shao Q Wright G Bader GD Morris Q The GeneMANIA prediction server: biological network integration for gene prioritization and predicting gene function Nucleic Acids Res 2010 38 Suppl 2 W214 W220 20576703 10.1093/nar/gkq537 PMC2896186

[B16] Bindschedler LV Palmblad M Cramer R Hydroponic isotope labelling of entire plants (HILEP) for quantitative plant proteomics; an oxidative stress case study Phytochemistry 2008 69 10 1962 1972 10.1016/j.phytochem.2008.04.007 18538804

[B17] Fernandez-Calvino L Faulkner C Walshaw J Saalbach G Bayer E Benitez-Alfonso Y Maule A Arabidopsis plasmodesmal proteome PLoS One 2011 6 4 e18880 10.1371/journal.pone.0018880 21533090 PMC3080382

[B18] Ito J Batth TS Petzold CJ Redding-Johanson AM Mukhopadhyay A Verboom R Meyer EH Millar AH Heazlewood JL Analysis of the arabidopsis cytosolic proteome highlights subcellular partitioning of central plant metabolism J Proteome Res 2011 10 4 1571 1582 10.1021/pr1009433 21166475

[B19] Mitra S Gantt J Ruby J Clouse S Goshe M Membrane proteomic analysis of arabidopsis thaliana using alternative solubilization techniques J Proteome Res 2007 6 5 1933 1950 10.1021/pr060525b 17432890

[B20] Kronegg J Buloz D Detection/prediction of GPI cleavage site (GPI-anchor) in a protein (DGPI) 1999 http://129.194.185.165/dgpi/

[B21] Schwacke R Fischer K Ketelsen B Krupinska K Krause K Comparative survey of plastid and mitochondrial targeting properties of transcription factors in Arabidopsis and rice Mol Genet Genomics 2007 277 6 631 646 10.1007/s00438-007-0214-4 17295027

[B22] Schwacke R Schneider A van der Graaff E Fischer K Catoni E Desimone M Frommer WB Flügge UI Kunze R ARAMEMNON, a novel database for arabidopsis integral membrane proteins Plant Physiol 2003 131 1 16 26 10.1104/pp.011577 12529511 PMC166783

[B23] Yilmaz A Mejia-Guerra MK Kurz K Liang X Welch L Grotewold E AGRIS: arabidopsis gene regulatory information server, an update Nucleic Acids Res 2011 39 D1118 D1122 10.1093/nar/gkq1120 21059685 PMC3013708

[B24] Zybailov B Rutschow H Friso G Rudella A Emanuelsson O Sun Q van Wijk KJ Sorting signals, N-terminal modifications and abundance of the chloroplast proteome PLoS One 2008 3 4 e1994 10.1371/journal.pone.0001994 18431481 PMC2291561

[B25] Book AJ Gladman NP Lee SS Scalf M Smith LM Vierstra RD Affinity purification of the Arabidopsis 26S proteasome reveals a diverse array of plant proteolytic complexes J Biol Chem 2010 285 33 25554 25569 10.1074/jbc.M110.136622 20516081 PMC2919120

[B26] Yang H Yang S Li Y Hua J The Arabidopsis BAP1 and BAP2 genes are general inhibitors of programmed cell death Plant Physiol 2007 145 135 146 10.1104/pp.107.100800 17631528 PMC1976577

[B27] Benschop JJ Mohammed S O’Flaherty M Heck AJ Slijper M Menke FL Quantitative phosphoproteomics of early elicitor signalling in Arabidopsis Mol Cell Proteomics 2007 6 7 1198 1214 10.1074/mcp.M600429-MCP200 17317660

[B28] Marmagne A Ferro M Meinnel T Bruley C Kuhn L Garin J Barbier-Brygoo H Ephritikhine G A high content in lipid-modified peripheral proteins and integral receptor kinases features the Arabidopsis plasma membrane proteome Mol Cell Proteomics 2007 6 11 1980 1996 10.1074/mcp.M700099-MCP200 17644812

[B29] Bayer EM Bottrill AR Walshaw J Vigouroux M Naldrett MJ Thomas CL Maule AJ Arabidopsis cell wall proteome defined using multidimensional protein identification technology Proteomics 2006 6 1 301 311 10.1002/pmic.200500046 16287169

[B30] Jaquinod M Villiers F Kieffer-Jaquinod S Hugouvieux V Bruley C Garin J Bourguignon J A proteomic dissection of Arabidopsis thaliana vacuoles isolated from cell culture Mol Cell Proteomics 2007 6 3 394 412 17151019 10.1074/mcp.M600250-MCP200 PMC2391258

[B31] Parsons HT Christiansen K Knierim B Carroll A Ito J Batth TS Smith-Moritz AM Morrison S McInerney P Hadi MZ Auer M Mukhopadhyay A Petzold CJ Scheller HV Loque D Heazlewood JL Isolation and proteomic characterization of the arabidopsis golgi defines functional and novel components involved in plant cell wall biosynthesis Plant Physiol 2012 159 1 12 26 10.1104/pp.111.193151 22430844 PMC3375956

[B32] Nikolovski N Rubtsov D Segura MP Miles GP Stevens TJ Dunkley TP Munro S Lilley KS Dupree P Putative glycosyltransferases and other plant Golgi apparatus proteins are revealed by LOPIT proteomics Plant Physiolin Press 10.1104/pp.112.204263 PMC3461528 22923678

[B33] Borderies G Jamet E Lafitte C Rossignol M Jauneau A Boudart G Monsarrat B Esquerré-Tugayé MT Boudet A Pont-Lezica R Proteomics of loosely bound cell wall proteins of Arabidopsis thaliana cell suspension cultures: a critical analysis Electrophoresis 2003 24 19–20 3421 3432 14595688 10.1002/elps.200305608

[B34] Lewis JD Lazarowitz SG Arabidopsis synaptotagmin SYTA regulates endocytosis and virus movement protein cell-to-cell transport Proc Natl Acad Sci USA 2010 107 6 2491 2496 10.1073/pnas.0909080107 20133785 PMC2823903

[B35] Yamazaki T Kawamura Y Minami A Uemura M Calcium-dependent freezing tolerance in Arabidopsis involves membrane resealing via synaptotagmin SYT1 Plant Cell 2008 20 12 3389 3404 10.1105/tpc.108.062679 19088330 PMC2630449

[B36] Minic Z Jamet E Négroni L Arsene der Garabedian P Zivy M Jouanin L A sub-proteome of Arabidopsis thaliana mature stems trapped on Concanavalin A is enriched in cell wall glycoside hydrolases J Exp Bot 2007 58 10 2503 2512 10.1093/jxb/erm082 17526915 PMC2394711

[B37] Oliviusson P Heinzerling O Hillmer S Hinz G Tse YC Jiang L Robinson DG Plant retromer, localized to the prevacuolar compartment and microvesicles in Arabidopsis, may interact with vacuolar sorting receptors Plant Cell 2006 18 1239 1252 10.1105/tpc.105.035907 16582012 PMC1456867

[B38] Jaillais Y Santambrogio M Rozier F Fobis-Loisy I Miège C Gaude T The retromer protein VPS29 links cell polarity and organ initiation in plants Cell 2007 130 1057 1070 10.1016/j.cell.2007.08.040 17889650

[B39] Kleine-Vehn J Leitner J Zwiewka M Sauer M Abas L Luschnig C Friml J Differential degradation of PIN2 auxin efflux carrier by retromer-dependent vacuolar targeting Proc Natl Acad Sci USA 2008 105 46 17812 17817 10.1073/pnas.0808073105 19004783 PMC2584678

[B40] Grienenberger E Besseau S Geoffroy P Debayle D Heintz D Lapierre C Pollet B Heitz T Legrand M A BAHD acyltransferase is expressed in the tapetum of Arabidopsis anthers and is involved in the synthesis of hydroxycinnamoyl spermidines Plant J 2009 58 2 246 259 10.1111/j.1365-313X.2008.03773.x 19077165

[B41] Dobritsa AA Geanconteri A Shrestha J Carlson A Kooyers N Coerper D Urbanczyk-Wochniak E Bench BJ Sumner LW Swanson R Preuss D A large-scale genetic screen in Arabidopsis to identify genes involved in pollen exine production Plant Physiol 2011 157 2 947 970 10.1104/pp.111.179523 21849515 PMC3192556

[B42] Brock AK Willmann R Kolb D Grefen L Lajunen HM Bethke G Lee J Nürnberger T Gust AA The Arabidopsis mitogen-activated protein kinase phosphatase PP2C5 affects seed germination, stomatal aperture, and abscisic acid-inducible gene expression Plant Physiol 2010 153 3 1098 1111 10.1104/pp.110.156109 20488890 PMC2899920

[B43] Schweighofer A Kazanaviciute V Scheikl E Teige M Doczi R Hirt H Schwanninger M Kant M Schuurink R Mauch F Buchala A Cardinale F Meskiene I The PP2C-type phosphatase AP2C1, which negatively regulates MPK4 and MPK6, modulates innate immunity, jasmonic acid, and ethylene levels in Arabidopsis Plant Cell 2007 19 7 2213 2224 10.1105/tpc.106.049585 17630279 PMC1955703

[B44] Guan Y Nothnagel EA Binding of arabinogalactan proteins by Yariv phenylglycoside triggers wound-like responses in Arabidopsis cell cultures Plant Physiol 2004 135 3 1346 1366 10.1104/pp.104.039370 15235117 PMC519053

[B45] Ding L Zhu J-K A role for arabinogalactan-proteins in root epidermal cell expansion Planta 1997 203 289 294 10.1007/s004250050194 9431677

[B46] Roy S Jauh GY Hepler PK Lord EM Effects of Yariv phenylglycoside on cell wall assembly in the Lily pollen tube Planta 1998 204 450 458 10.1007/s004250050279 9684368

[B47] Molle J-C Kim S Jauh Y Lord EM Arabinogalactan proteins, pollen tube growth, and the reversible effect of Yariv phenylglycoside Protoplasma 2002 219 89 98 10.1007/s007090200009 11926071

[B48] Nguema-Ona E Banningan A Chevalier L Baskin TI Driouich A Disruption of arabinogalactan proteins disorganizes cortical microtubules in the root of Arabidopsis thaliana Plant J 2007 52 240 251 10.1111/j.1365-313X.2007.03224.x 17672840

[B49] Kipreos ET Pagano M The F-box protein family Genome Biol 2000 1 5 R3002 10.1186/gb-2000-1-5-reviews3002 PMC138887 11178263

[B50] Xu G Ma H Nei M Kong H Evolution of F-box genes in plants:different modes of sequence divergence and their relationships with functional diversification Proc Natl Acad Sci USA 2009 106 835 840 10.1073/pnas.0812043106 19126682 PMC2630105

[B51] Skaar JR Pagan JK Pagano M SnapShot: F-box proteins I Cell 2009 137 1160 1161 10.1016/j.cell.2009.05.039 19524517

[B52] Hua Z Zou C Shiu S-H Vierstra RD Phylogenetic comparison of F-Box (FBX) superfamily within the plant kingdom reveals divergent evolutionary histories indicative of genomic drift PLoS One 2011 6 e16219 10.1371/journal.pone.0016219 21297981 PMC3030570

[B53] Gusti A Baumberger N Nowack M Pusch S Eisler H Potuschak T De Veylder L Schnittger A Genschik P The Arabidopsis thaliana F-Box protein FBL17 is essential for progression through the second mitosis during pollen development PLoS One 2009 4 3 e4780 10.1371/journal.pone.0004780 19277118 PMC2651519

[B54] Doelling JH Phillips AR Soyler-Ogretim G Wise J Chandler J Callis J Otegui MS Vierstra RD The ubiquitin-specific protease subfamily UBP3/UBP4 is essential for pollen development and transmission in Arabidopsis Plant Physiol 2007 145 801 813 10.1104/pp.106.095323 17905865 PMC2048767

[B55] Dobney S Chiasson D Lam P Smith SP Snedden WA The calmodulin-related calcium sensor CML42 plays a role in trichome branching J Biol Chem 2009 284 46 31647 31657 10.1074/jbc.M109.056770 19720824 PMC2797235

[B56] Popescu SC Popescu GV Bachan S Hang Z Seay M Gerstein M Snyder M Dinesh-Kumar SP Differential binding of calmodulin-related proteins to their targets revealed through high-density Arabidopsis protein microarrays Proc Natl Acad Sci USA 2007 104 11 4730 4735 10.1073/pnas.0611615104 17360592 PMC1838668

[B57] Schiøtt M Romanowsky SM Bækgaard L Jakobsen MK Palmgren MG Harper J A plant plasma membrane Ca2+ pump is required for normal pollen tube growth Proc Natl Acad Sci USA 2004 101 9502 9507 10.1073/pnas.0401542101 15197266 PMC439006

[B58] Allen AM Lexer C Hiscock SJ Comparative analysis of pistil transcriptomes reveals conserved and novel genes expressed in dry, wet and semidry stigmas Plant Physiol 2010 154 1347 1360 10.1104/pp.110.162172 20813907 PMC2971611

[B59] Boavida LC Borges F Becker JD Feijó JA Whole genome analysis of gene expression reveals coordinated activation of signaling and metabolic pathways during pollen-pistil interactions in Arabidopsis Plant Physiol 2011 155 2066 2080 10.1104/pp.110.169813 21317340 PMC3091125

[B60] Elleman CJ Dickinson HG Commonalities between pollen/stigma and host/pathogen interactions: Calcium accumulation during stigmatic penetration by Brassica oleracea pollen tubes Sex Plant Reprod 1999 12 194 202 10.1007/s004970050192

[B61] Gibbs GM Roelants K O’Bryan MK The CAP superfamily: Cysteine-rich secretory proteins, Antigen 5, and Pathogenesis-related 1 proteins — roles in reproduction, cancer, and immune defense Endocr Rev 2008 29 865 897 10.1210/er.2008-0032 18824526

[B62] Gibbs GM Lo JCY Nixon B Jamsai D O’Connor AE Rijal S Sanchez-Partida LG Hearn MTW Bianco DM O’Bryan MK Glioma pathogenesis-related 1-like 1 is testis enriched, dynamically modified, and redistributed during male germ cell maturation and has a potential role in sperm-oocyte binding Endocrinology 2010 151 2331 2342 10.1210/en.2009-1255 20219979

[B63] Ohnishi T Takanashi H Mogi M Takahashi H Kikuchi S Yano K Okamoto T Fujita M Kurata N Tsutsumi N Distinct gene expression profiles in egg and synergid cells of rice as revealed by cell type-specific microarrays Plant Physiol 2011 155 881 891 10.1104/pp.110.167502 21106719 PMC3032473

[B64] Kessler SA Shimosato-Asano H Keinath NF Wuest SE Ingram G Panstruga R Grossniklaus U Conserved Molecular Components for Pollen Tube Reception and Fungal Invasion Science 2010 330 6006 968 971 10.1126/science.1195211 21071669

[B65] Govers F Angenent GC Fertility goddesses as trojan horses Science 2010 330 6006 922 923 10.1126/science.1198347 21071655

[B66] Rotin D Staub O Haguenauer-Tsapis R Ubiquitination and endocytosis of plasma membrane proteins:role of Nedd4/Rsp5p family of ubiquitin-protein ligases J Membr Biol 2000 176 1 17 10882424 10.1007/s00232001079

[B67] Dunn R Klos DA Adler AS Hicke L The C2 domain of the Rsp5 ubiquitin ligase binds membrane phosphoinositides and directs ubiquitination of endosomal cargo J Cell Biol 2004 165 1 135 144 10.1083/jcb.200309026 15078904 PMC2172079

[B68] Šamaj J Read ND Volkmann D Menzel D Baluska F The endocytic network in plants Trends Cell Biol 2005 15 425 433 10.1016/j.tcb.2005.06.006 16006126

[B69] Robinson DG Jiang L Schumacher K The endosomal system of plants: Charting new and familiar territories Plant Physiol 2008 147 1482 1492 10.1104/pp.108.120105 18678740 PMC2492610

[B70] Zhao Y Yan A Feijó JA Furutani M Takenawa T Hwang I Fu Y Yang Z Phosphoinositides regulate clathrin-dependent endocytosis at the tip of pollen tubes in Arabidopsis and tobacco Plant Cell 2010 22 12 4031 4044 10.1105/tpc.110.076760 21189293 PMC3027160

[B71] Herman EM Lamb CJ Arabinogalactan-rich glycoproteins are localized on the cell surface and in intravacuolar multivesicular bodies Plant Physiol 1992 98 264 272 10.1104/pp.98.1.264 16668623 PMC1080178

[B72] Šamaj J Šamajová O Peters M Baluška F Lichtscheidl I Knox JP Volkmann D Immunolocalization of LM2 arabinogalactan-protein epitope associated with endomembranes of plant cells Protoplasma 2000 212 186 196 10.1007/BF01282919

[B73] Dettmer J Hong-Hermesdorf A Stierhof YD Schumacher K Vacuolar H+-ATPase activity is required for endocytic and secretory trafficking in Arabidopsis Plant Cell 2006 18 715 730 10.1105/tpc.105.037978 16461582 PMC1383645

[B74] Schumacher K Vafeados D McCarthy M Sze H Wilkins T Chory J The Arabidopsis det3 mutant reveals a central role for the vacuolar H(+)-ATPase in plant growth and development Genes Dev 1999 13 3259 3270 10.1101/gad.13.24.3259 10617574 PMC317205

[B75] Catz SD Johnson JL Babior BM The C2A domain of JFC1 binds to 3′-phosphorylated phosphoinositides and directs plasma membrane association in living cells Proc Natl Acad Sci USA 2002 99 18 11652 11657 10.1073/pnas.172382799 12189202 PMC129324

[B76] Yang H Li Y Hua J The C2 domain protein BAP1 negatively regulates defense responses in Arabidopsis Plant J 2006 48 238 248 10.1111/j.1365-313X.2006.02869.x 17018034

[B77] Lee CB Swatek KN McClure B Pollen proteins bind to the C-terminal domain of Nicotiana alata pistil arabinogalactan proteins J Biol Chem 2008 283 26965 26973 10.1074/jbc.M804410200 18678868

[B78] Pourcher M Santambrogio M Thazar N Thierry AM Fobis-Loisy I Miège C Jaillais Y Gaude T Analyses of sorting nexins reveal distinct retromer-subcomplex functions in development and protein sorting in Arabidopsis thaliana Plant Cell 2010 22 12 3980 3991 10.1105/tpc.110.078451 21156856 PMC3027177

[B79] Bove J Vaillancourt B Kroeger J Hepler PK Wiseman PW Geitmann A Magnitude and direction of vesicle dynamics in growing pollen tubes using spatiotemporal image correlation spectroscopy (STICS) and fluorescence recovery after photobleaching (FRAP) Plant Physiol 2008 147 4 1646 1658 10.1104/pp.108.120212 18508956 PMC2492615

[B80] Bosch M Hepler PK Pectin methylesterases and pectin dynamics in pollen tubes Plant Cell 2005 17 12 3219 3226 10.1105/tpc.105.037473 16322606 PMC1315365

[B81] Kuromori T Hirayama T Kiyosue Y Takabe H Mizukado S Sakurai T Akiyama K Kamiya A Ito T Shinozaki K A collection of 11 800 single-copy Ds transposon insertion lines in Arabidopsis Plant J 2004 37 897 905 10.1111/j.1365.313X.2004.02009.x 14996221

[B82] Dardelle F Lehner A Ramdani Y Bardor M Lerouge P Driouich A Mollet JC Biochemical and immunocytological characterizations of Arabidopsis pollen tube cell wall Plant Physiol 2010 153 4 1563 1576 10.1104/pp.110.158881 20547702 PMC2923879

[B83] Li C Wong HW Model-based analysis of oligonucleotide arrays:expression index computation and outlier detection Proc Natl Acad Sci USA 2001 98 31 36 10.1073/pnas.98.1.31 11134512 PMC14539

[B84] Li C Wong HW Model-based analysis of oligonucleotide arrays: model validation, design issues and standard error application Genome Biol 2001 2 8 R32 10.1186/gb-2001-2-8-research0032 PMC55329 11532216

[B85] Livak KJ Schmittgen TD Analysis of relative gene expression data using real-time quantitative PCR and the 2(−Delta Delta C(T)) method Methods 2001 25 4 402 408 10.1006/meth.2001.1262 11846609

[B86] Gietz D St. Jean A Woods RA Schiestl RH Improved method for high efficiency transformation of intact yeast cells Nucleic Acids Res 1992 20 1425 10.1093/nar/20.6.1425 1561104 PMC312198

[B87] Sherman F Getting started with yeast Methods Enzymol 2002 350 3 41 12073320 10.1016/s0076-6879(02)50954-x

