# Supplementary material for: Expression-based and co-localization detection of arabinogalactan protein 6 and arabinogalactan protein 11 interactors in Arabidopsis pollen and pollen tubes
Source: BMC Plant Biol. 2013 Jan 8;13:7. doi: 10.1186/1471-2229-13-7 (PMC3546934; doi:10.1186/1471-2229-13-7)
Supplement: Additional file 2 — Differentially expressed genes common to agp6 agp11 mutant and treatment with Yariv phenylglycoside reagent for 1 h and 10 h. [file 1471-2229-13-7-S2.docx]

| Additional file 2. *Differentially expressed genes common to agp6 agp11 mutant and treatment with Yariv phenylglycoside reagent for 1h and 10 h* | | | | |  |
| --- | --- | --- | --- | --- | --- |
| AGI locus | Fold change^a^ | | | Gene Description^c^ | |
|  | agp6 agp11 | Yariv  1h^b^ | Yariv  10h^b^ |  |  |
| At3g57880 | +25.3 |  | +2.64 | T10K17.90 Calcium-dependent lipid-binding (CaLB domain) plant phosphoribosyltransferase family protein | |
| At5g48540 | +14.9 | +3.48 |  | MJE7.18 receptor-like protein kinase-related family protein | |
| At1g23020 | +2.97 |  | +2.83 | FRO3 ferric-chelate reductase | |
| At5g05110 | +2.67 |  | +2.30 | Cystatin; monellin family protein | |
| At3g55890 | +2.38 |  | +2.30 | F27K19.70 yippee family putative zinc-binding protein | |
| At2g40000 | +3.34 | +4.59 |  | HSPRO2 ortholog of sugar beet HS1 PRO-1 2 | |
| At3g60130 | +1.96 |  | +3.25 | BGLU16 beta glucosidase 16 | |
| At1g53580 | +2.42 |  | +2.30 | ETHE1 Mononuclear Fe(II)-containing member of the b-lactamase fold superfamily; GLY3 glyoxalase II3 | |
| At2g44790 | +4.18 | +3.25 | +4.29 | UCC2 uclacyanin; copper ion binding | |
| At3g61450 | +1.89 |  | +4.29 | SYP73 syntaxin of plants 73 | |
| At4g20780 | +1.90 | +2.64 |  | Calmodulin-like 42; Calcium sensor involved in trichome branching | |
| At4g37370 | +2.73 |  | +3.03 | CYP81D8 member of CYP81D; oxygen binding -- cytochrome P450 | |
| At5g42380 | +1.74 | +16.0 |  | Calmodulin-like 37 | |
| At3g50930 | +2.08 | +2.64 |  | BCS1 cytochrome BC1 synthesis | |
| At4g37260 | +1.67 | +2.14 |  | MYB73 Member of the R2R3 factor gene family; | |
| At3g27820 | +1.62 |  | +2.00 | MDAR4 peroxisome membrane-bound monodehydroascorbate reductase | |
| At1G53540 | -1.83 |  | -2.05 | HSP20-like chaperones superfamily protein | |
| At5g59960 | -1.60 |  | -2.08 | Unknown protein | |
| At3g47810 | -1.56 | -2.17 |  | MAG1 Homolog of yeast retromer subunit VPS29 | |
| At2g03020 | -1.91 |  | -2.13 | Heat shock protein-related HSP20 ; alpha crystallin family | |
| At4g14615 | -2.02 | -2.05 |  | Unknown protein | |
| At1g78260 | -3.48 |  | -2.74 | RNA-binding (RRM/RBD/RNP motifs) family protein | |
| At1g59860 | -2.78 |  | -2.12 | HSP20-like chaperones superfamily protein | |
| At5g12030 | -2.99 |  | -2.80 | HSP17.6 cytosolic small heat shock protein with chaperone activity | |
| At3G46230 | -2.62 |  | -3.3 | HSP17.4 member of the class I small heat-shock protein (sHSP) family | |
| At3G15210 | -2.77 | -2.14 |  | ERF4 ethylene response factor, subfamily B-1 of ERF/AP2 transcription factor family | |
| At4g04800 | -3.17 | -2.39 | -2.06 | MSRB3: methionine sulfoxide reductase domain-containing protein | |
| At5g52420 | -6.87 |  | -2.19 | Unknown protein | |
| At2g29500 | -8.22 |  | -2.90 | HSP20-like chaperones superfamily protein | |
| ^a^ Genes up or down regulated are indicated with a (+) or a (-) sign respectively.  ^b^ Data from [14]. Blanks in the table indicate that the change in gene expression was less than a 2-fold increase or decrease  ^c^ TAIR gene annotations [61]. | | | | | |
